# Uncovering an Unusual FBN1 Gene Mutation Responsible for Marfan Syndrome: A Case Study

**DOI:** 10.7759/cureus.59452

**Published:** 2024-05-01

**Authors:** Gabriel A Jiménez-Berríos, Sebastián J Vázquez-Folch, Natalio Izquierdo

**Affiliations:** 1 School of Medicine, Universidad Central del Caribe, Bayamón, PRI; 2 Department of Surgery, School of Medicine, Medical Sciences Campus, University of Puerto Rico, San Juan, PRI

**Keywords:** marfan syndrome, cysteine, fibrillin-1, autosomal dominant inheritance, ectopia lentis

## Abstract

Patients with Marfan syndrome have a constellation of clinical features and a heterogeneous phenotype. The purpose of this study is to report a 47-year-old male patient with an unusual variant in the *FBN1 *gene causing Marfan syndrome. The patient with musculoskeletal, cardiovascular, and ocular findings compatible with Marfan syndrome had an unusual pathogenic mutation on the *FBN1* gene. The patient was examined by at least one of the authors (NJI). The patient’s clinical findings were compatible with Marfan syndrome. Our patient had a unique mutation in the *FBN1* gene (c.8054A>G p.His2685Arg) located on exon 65. Next-generation sequencing was done using the Invitae panel. This variant was categorized as one of uncertain significance. This patient’s variant on the *FBN1* gene leading to the syndrome has scant data associated with it and this is the first time it is reported from Puerto Rico.

## Introduction

Marfan syndrome (MFS) presents a range of clinical features and a diverse phenotype, with diagnosis primarily relying on the revised Ghent criteria [1] and genetic findings. Clinical systemic manifestations include musculoskeletal [2], cardiovascular, and ophthalmic manifestations. Musculoskeletal signs include tall stature, extended limbs, arachnodactyly, dolichostenomelia, joint hypermobility, chest deformities such as pectus excavatum (sunken chest) or pectus carinatum (pigeon chest), underdeveloped upper jaw (maxillary hypoplasia), and a high-arched palate often referred to as a gothic palate [3]. The cardiovascular issues associated with the syndrome encompass enlargement and splitting of the aortic root, along with mitral valve prolapse and leakage [4]. Ophthalmological complications include lens dislocation, strabismus, glaucoma, and retinal detachment [5,6].

MFS is inherited as an autosomal dominant trait. Dietz first described the *FBN1* gene associated with the syndrome [7]. There are over 2,000 published *FBN1* variants, and many are unique to individual families [8]. Missense variants, especially cysteine substitutions, are the most common type of *FBN1* variant [9]. The type of *FBN1* variant identified and the likelihood of that variant being pathogenic are recognized as important factors when diagnosing MFS, with de novo (in the absence of family history), nonsense, frameshift, splicing, and missense substitutions of conserved residues considered most likely to be pathogenic [9]. Identifying pathogenic or likely pathogenic variants in the *FBN1* gene, linked with specific clinical manifestations such as aortic root enlargement or lens dislocation, plays a crucial role in diagnosing MFS [10].

Cysteine residues are pivotal for fibrillin-1 structure, a protein essential for connective tissue integrity, with missense mutations often disrupting protein folding and leading to clinical manifestations [11]. The structure of fibrillin-1 is distinguished by its modular domain organization, featuring two types of cysteine-rich domains that repeat throughout its sequence [11]. Our discovery of an unusual variant in a patient, characterized by the substitution of histidine for arginine, diverges from the common theme of cysteine-related mutations in MFS. This finding is noteworthy because it does not involve a cysteine mutation, thereby suggesting a different mechanism of disease manifestation and highlighting the complexity of genotype-phenotype relationships in MFS.

Here, we report the case of a patient with an unusual variant in the *FBN1* gene who had systemic manifestations and ectopia lentis as part of the syndrome. This is the first report of this variant in the Puerto Rican population.

## Case presentation

A 47-year-old male patient from Puerto Rico was referred for ophthalmic evaluation by his cardiologist. The patient had a history of heart disease, smoking, hypertension, and asthma. Systemic medications included carvedilol and warfarin. Upon physical examination, the patient had long extremities, dolichostenomelia, positive wrist and ulnar signs, arachnodactyly, malar hypoplasia, and gothic palate. The patient had a surgical history of descending aorta repair.

**Figure 1:**
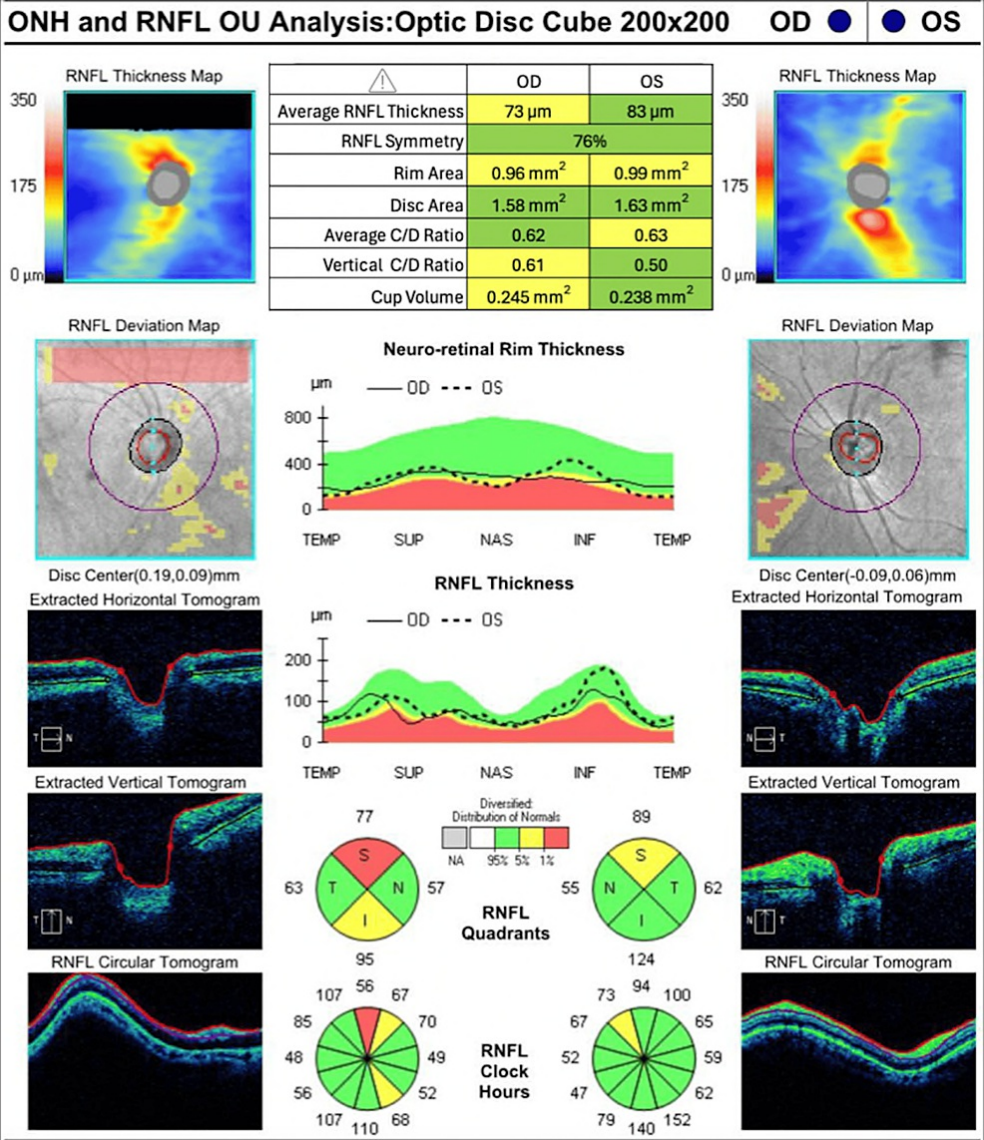
Optic nerve coherence tomography. The most recent optical coherence tomography of the patient demonstrates the asymmetry between both eyes in the retinal nerve fiber layer. Row 1: Average retinal nerve fiber layer thickness. Row 2: Retinal nerve fiber layer symmetry. Row 3: Rim area. Row 4: Disc area. Row 5: Average cup/disc ratio. Row 6: Vertical cup/disc ratio. Row 7: Cup volume.

Upon comprehensive ophthalmic evaluation by at least one of the authors (NJI), the best-corrected visual acuity was 20/50 and 20/30 in the right and left eye, respectively. Refraction was -11.50 +5.50 × 120° and -7.00 +4.50 × 70° in the right and left eye, respectively. Upon slit-lamp examination (SLE), the patient had a smooth velvety iris without iridodonesis and lens subluxation. SLE also revealed white and quiet sclera OU and a clear lens bilaterally. Upon indirect ophthalmoscopy, the patient had asymmetric cupped optic nerves, intact vessels, maculae, and peripheries. Upon optic nerve coherence tomography (Carl Zeiss Meditec, Inc.), the patient had a retinal fiber layer of 73 µm and 83 µm, and the average cup-to-disk ratio was 0.62 and 0.63 in the right and left eye, respectively (Figure 1).

Visual field testing (30-2 Carl Zeiss Meditec, Inc.) showed a mean deviation of -8.96 dB (p < 0.5%) and -2.65 dB (p < 0.5%) in the right and left eye, respectively.

The patient was diagnosed with glaucoma. He was treated with brimonidine 0.15% drops, one drop in both eyes three times daily (TID). The patient’s condition is currently stable.

*FBN1* full gene sequencing was done (Laboratory for Molecular Medicine, Center for Genetics and Genomics, Cambridge, MA). It showed a heterozygous mutation in the *FBN1* gene with a novel presumed pathogenic variant (c.8054A>G p.His2685Arg) on exon 65.

## Discussion

Past literature has reported that patients with MFS have several musculoskeletal findings [1]. Our patient had pectus excavatum, long extremities, dolichostenomelia, positive wrist sign, positive ulnar sign, and arachnodactyly. These findings are compatible with previous literature [1,3,7].

Ziegler et al. have reported different cardiovascular manifestations present in MFS. These include aortic root dilation and dissection, mitral valve prolapse, and aortic insufficiency [12]. Our patient had aortic root dissection. His cardiovascular findings were compatible with MFS.

Ocular observations in individuals with MFS have been thoroughly documented [13]. Our patient had myopia, smooth velvety iris, ectopia lentis, and optic nerve cups. These findings are compatible with previous studies.

Glaucoma has been reported in patients with the syndrome [5,13]. Both optic nerve cup asymmetry and visual field testing results were compatible with glaucoma in this patient. Glaucoma evaluation in all patients with MFS is needed.

Our patient had a unique mutation in the *FBN1* gene (c.8054A>G p.His2685Arg) located on exon 65. This particular variant has been classified as of uncertain significance [14]. The discovery of this mutation not only contributes a novel aspect to the genetic understanding of MFS but also suggests that the genetic mechanisms underlying certain eye-related symptoms of the syndrome could be more complex than what is currently understood.

## Conclusions

We report the case of a patient whose clinical manifestations were compatible with MFS, despite having a variant of unknown significance in the *FBN1* gene. The association of such a pathogenic phenotypic characteristic to this variant (c.8054A>G p.His2685Arg) on exon 65 emphasizes the critical need to evaluate all variants within a gene. This approach not only enhances our understanding of the molecular foundation of MFS but also paves the way for more personalized treatment options, ultimately broadening the scope of therapeutic strategies for this condition.
